# *Schistosoma mansoni* Eggs Modulate the Timing of Granuloma Formation to Promote Transmission

**DOI:** 10.1016/j.chom.2020.10.002

**Published:** 2021-01-13

**Authors:** Kevin K. Takaki, Gabriel Rinaldi, Matthew Berriman, Antonio J. Pagán, Lalita Ramakrishnan

**Affiliations:** 1 Molecular Immunity Unit, Department of Medicine, University of Cambridge, MRC Laboratory of Molecular Biology, Cambridge CB2 0QH, UK; 2 Wellcome Sanger Institute, Wellcome Genome Campus, Hinxton CB10 1SA, UK

**Keywords:** schistosomiasis, *Schistosoma mansoni*, zebrafish, humans, granulomas, innate immunity, Schistosoma egg extrusion, Schistosoma egg translocation, miracidia, helminths

## Abstract

Schistosome eggs provoke the formation of granulomas, organized immune aggregates, around them. For the host, the granulomatous response can be both protective and pathological. Granulomas are also postulated to facilitate egg extrusion through the gut lumen, a necessary step for parasite transmission. We used zebrafish larvae to visualize the granulomatous response to *Schistosomamansoni* eggs and inert egg-sized beads. Mature eggs rapidly recruit macrophages, which form granulomas within days. Beads also induce granulomas rapidly, through a foreign body response. Strikingly, immature eggs do not recruit macrophages, revealing that the eggshell is immunologically inert. Our findings suggest that the eggshell inhibits foreign body granuloma formation long enough for the miracidium to mature. Then parasite antigens secreted through the eggshell trigger granulomas that facilitate egg extrusion into the environment. In support of this model, we find that only mature *S. mansoni* eggs are shed into the feces of mice and humans.

## Highlights

• Foreign bodies are walled off by immune structures called granulomas
• *Schistosoma mansoni* eggshells prevent the formation of granulomas around immature parasites
• Secreted antigens from mature parasites induce granulomas that promote egg shedding
• *S. mansoni* modulates granuloma formation to selectively shed mature eggs into feces

## Graphical Abstract

**Figure:**
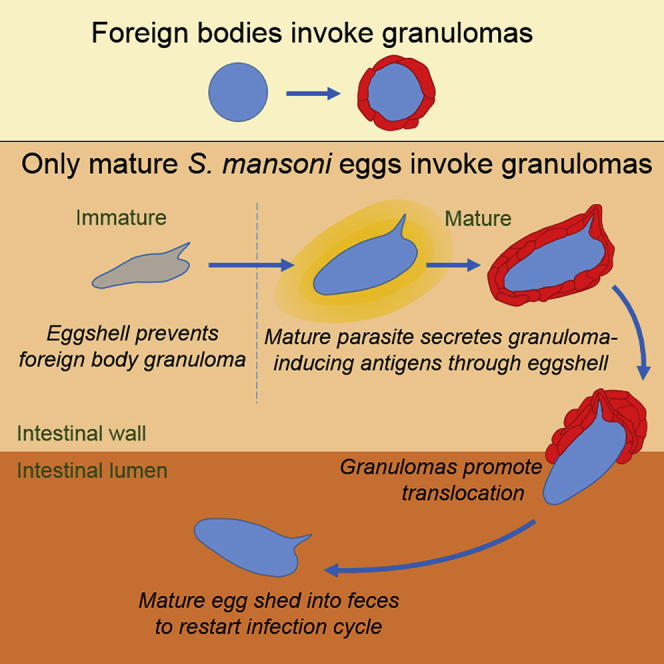

## Teaser

Using a zebrafish model, Takaki et al. show that the *Schistosoma mansoni* eggshell acts as an immunologically inert barrier, preventing the formation of granulomas around immature eggs. Mouse and human data support the model that secreted antigens from maturing parasites stimulate granulomas to selectively shed only mature eggs into the environment to restart their life cycle.

## Introduction

Human schistosomiasis, caused by parasitic flatworms of the genus *Schistosoma*, affects more than 200 million people worldwide (WHO, 2019). Adult schistosomes live in the mesenteric venules of their definitive hosts, humans, where they produce eggs that are shed into the environment through feces or urine, depending on the schistosome species (Colley and Secor, 2014). Upon reaching fresh water, the eggs hatch releasing free-swimming larvae, miracidia, that can then infect their intermediate snail hosts (Colley and Secor, 2014). In the snails, they reproduce asexually and mature to produce cercarial larvae, which are released into the water, and infect humans by penetrating the skin (Colley and Secor, 2014). In the case of *Schistosoma mansoni*, the most studied and geographically widespread species, the egg-laying adult pair resides in the mesenteric venous plexus. Upon maturation in the liver, the female and male adult worms pair up and migrate via the portal system to the mesenteric venules where they produce eggs (Nation et al., 2020). The eggs are shed by translocation through the venule and then the intestinal wall into the feces; however, many become lodged in the intestinal wall or the liver (Hams et al., 2013; McManus et al., 2018; Nation et al., 2020; Schwartz and Fallon, 2018).

As the egg matures, it secretes antigens that provoke the formation of a granuloma—an organized aggregate of macrophages and other immune cells—around it (Ashton et al., 2001; Boros and Warren, 1970; Chiu and Chensue, 2002; Jurberg et al., 2009). For the host, the granuloma may play a dual function—both protective and pathogenic (Hams et al., 2013). On the one hand, it may protect the host by sequestering toxic egg antigens and by preventing translocation of bacteria from the intestinal lumen into the tissues as the egg breaches the intestinal wall to exit the host (Costain et al., 2018; Hams et al., 2013; Pagán and Ramakrishnan, 2018; Schwartz and Fallon, 2018). On the other hand, the chronic granulomas around tissue-trapped eggs, particularly those in the liver, are the principal drivers of disease pathogenesis and morbidity (Hams et al., 2013; Pagán and Ramakrishnan, 2018). The chronic *Schistosoma* granuloma has a complex cellular composition with an abundance of myeloid cells, lymphocytes, eosinophils, and fibroblasts that act in concert to cause tissue pathology (Hams et al., 2013; Pagán and Ramakrishnan, 2018). The fibrogenic granulomatous response to the liver-trapped eggs causes damaging periportal fibrosis leading to portal hypertension and the development of esophageal varices that can rupture, leading to internal bleeding and death (Colley and Secor, 2014; Pagán and Ramakrishnan, 2018).

While the granuloma’s role has mainly been studied from a host-centric view, it has also been hypothesized that the early granuloma is critical for the parasite’s life cycle by facilitating the translocation of the eggs from the vasculature to the intestines and then into the feces for transmission to a new host (Dunne et al., 1983; Hams et al., 2013; Schwartz and Fallon, 2018). Because insights into the *Schistosoma* granuloma have been derived from single time point histologic studies of human clinical samples and animal models—hamsters, mice, and monkeys (Cheever et al., 2002; Hutchison, 1928), its role in translocation is understudied. The optical transparency of the zebrafish larva has enabled detailing of the early events of tuberculous granuloma formation in real time using non-invasive, high-resolution, serial intravital microscopy (Pagán and Ramakrishnan, 2018; Ramakrishnan, 2020; Takaki et al., 2013). Here, we have used the zebrafish larva to detail the events of early granuloma formation to *S. mansoni* eggs. We find that macrophage-dense epithelioid granulomas form rapidly around mature eggs. In striking contrast, we find that immature eggs are immunologically silent, failing to provoke even minimal macrophage recruitment. Given that inert beads induce epithelioid granulomas, this finding provides insight into how the egg might actively manipulate the timing of granuloma formation so as to prevent immune destruction or premature extrusion from the host. This idea is supported by our findings that *S. mansoni*-infected mice have both mature and immature eggs in their liver and intestinal wall but shed only mature eggs into the intestinal lumen.

## Results

### *S. mansoni* Eggs Induce Epithelioid Granuloma Formation in the Context of Innate Immunity

**Figure 1:**
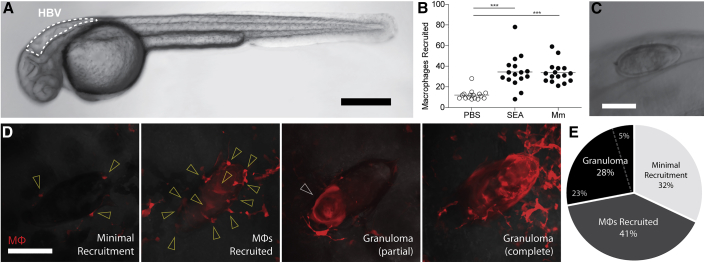
Macrophage Responses to SEA and *S. mansoni* Eggs (A) Zebrafish larvae at 30 h post fertilization (hpf) with hindbrain ventricle (HBV) outlined. Scale bar, 300 μm. (B) Mean macrophage recruitment to HBV 3 h post-injection with phosphate-buffered saline (PBS), SEA, or *Mycobacterium marinum* (Mm); ANOVA with Dunnett’s post-test. (C) *S. mansoni* egg in HBV immediately after implantation. Scale bar, 75 μm. (D) Representative images of macrophage responses to *S. mansoni* eggs observed 5 dpi; minimal recruitment, few if any macrophages recruited with ≤6 in contact with the egg (arrowheads); macrophages recruited, several macrophages recruited with >6 in contact with the egg (arrowheads) but without aggregation; granuloma; macrophage aggregation in which individual macrophages cannot be distinguished as separate, either partially (arrowhead) or completely encasing the egg. Scale bar, 75 μm. (E) Prevalence of macrophage responses to implanted eggs as defined in (D), representing 8 experiments, each constituting a separate batch of eggs and a separate clutch of zebrafish larvae, as detailed in Table S1. Dotted line divides the proportion of partial (23%) and complete (5%) granulomas. Also see Figure S1; Table S1; Video S1.

To study *Schistosoma* granulomas we used the zebrafish hindbrain ventricle (HBV), an epithelium-lined cavity to which phagocytes are recruited in response to chemokines and bacteria (Cambier et al., 2017; Cambier et al., 2014; Takaki et al., 2013; Yang et al., 2012) (Figure 1A). It has previously been shown that beads coated with *S. mansoni* soluble egg antigens (SEAs) injected intravenously into mice get deposited in the lung where they induce macrophage recruitment and aggregation around them (Boros and Warren, 1971; Chiu et al., 2004). Using transgenic zebrafish with red-fluorescent macrophages, we found that injection of SEA into the HBV induced macrophage recruitment within 6 h (Figure 1B). Next, we implanted *S. mansoni* eggs into the HBV. Because the mature egg is relatively large (>50-μm diameter), we used a large bore borosilicate needle that allowed us to make an incision, grasp the egg, and implant it into the HBV cavity in rapid succession (Figure S1; Video S1 and STAR Methods, Figure 1C). Implantation of the eggs had no deleterious effect on larval survival; larvae implanted with either one or two eggs had a survival rate of 98%–100% at 5 days post-implantation (dpi), identical to the mock-implanted control group (n = 50 per group). Implantation also did not change larval swimming behaviors or responses to tactile stimuli.

Video S1. Schistosome Egg Implantation, Related to Figure S1 and STAR Methods

We examined macrophage responses to the egg at 5 dpi. Eight independent experiments showed a consistent pattern of varying levels of macrophage recruitment: some eggs (32%, range 17% to 44%) had minimal macrophage recruitment with 0–6 macrophages found in contact with the egg (Figures 1D and 1E; Table S1). The majority (69%, range 56% to 83%) elicited robust macrophage recruitment with 41% (range 11% to 67%) having several isolated macrophages or small clusters of macrophages in contact with them and 28% (0% to 45%) eliciting organized granulomas that had either partially or fully enveloped them (Figures 1D and 1E; Table S1).

**Figure 2:**
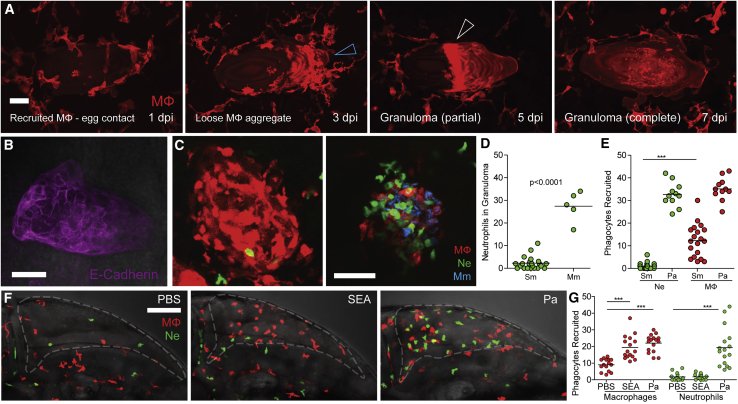
*S. mansoni* Eggs Induce Epithelioid Granulomas in Larval Zebrafish (A) Time-lapse microscopy of egg monitored at 2-day intervals from 1–7 dpi showing in the four panels, respectively, sequential macrophage recruitment, aggregation (blue arrowhead), formation of the partial granuloma (white arrowhead), and its expansion to encase the egg. Scale bar, 25 μm. (B) Epithelioid granuloma immunostained using E-cadherin antibody. Scale bar, 50 μm. (C) Confocal images of granulomas in representative transgenic zebrafish larvae with red-fluorescent macrophages (MΦ) and green-fluorescent neutrophils (Ne) at 5 dpi with *S. mansoni* eggs (Sm) (left) or 5 days post-infection with *M. marinum* (Mm) (right). Scale bar, 50 μm. (D) Quantification of neutrophils recruited to Sm and Mm granulomas. (E) Quantification of phagocytes recruited to Sm and *P. aeruginosa* (Pa) 6 h post-injection. (F) Confocal images of HBV of representative larvae showing phagocyte recruitment at 6 h post-injection with phosphate buffered saline (PBS) (left), *S. mansoni* SEA or *P.aeruginosa*. Scale bar, 100 μm. (G) Quantification of phagocytes recruited to Sm and *P.aeruginosa* (Pa) at 6 h post-injection. Horizontal lines in (D, E, and G) depict mean values. Student’s t test (D) or one-way ANOVA with Bonferroni's post-test (E and G). Experiments in (A, B, and E) were done once each, those in (C, D, F, and G) are representative of two experiments. Also see Figures S2 and S3; Video S2.

To determine the macrophage recruitment events leading to granuloma formation, we imaged nine implanted eggs sequentially over 7 days, and then analyzed retrospectively the progression of recruitment in the three that had formed granulomas (Figures 2A and S2). For the egg shown in Figure 2A, by 1 dpi, macrophages had arrived in response to the egg and were in contact with it (Figure 2A; Video S2). By 3 dpi, macrophages had formed loose aggregates on one part of the egg (Figure 2A), a transient stage that is likely to represent a transition to granuloma formation as it was not seen in our 5 dpi single timepoint analyses. By 5 dpi, an organized granuloma partially covering the egg was apparent, which had expanded to encapsulate the entire egg by 7 dpi (Figure 2A; Video S2). In the remaining two eggs that elicited granulomas, one had a similar sequence of events except that the granuloma, which formed by 5 dpi, had still not enveloped the egg completely at 7 dpi (Figure S2A). The other egg had already formed a small partial granuloma by 3 dpi but could not be monitored further owing to failure to recover the animal following imaging on this day (Figure S2B). Thus, the sequence of events leading to granuloma formation seemed consistent in all cases.

Video S2. Formation of the Schistosome Egg Granuloma, Related to Figure 2

In all three cases, even the partial granulomas had macrophages that appeared confluent with indistinct intercellular boundaries, suggesting that they had already undergone the characteristic epithelioid transformation associated with mature *Schistosoma* granulomas (Moore et al., 1977; Von Lichtenberg et al., 1973) (Figures 2A and S2). To confirm this, we identified 8 eggs that had elicited partial or complete granulomas and assessed them for epithelioid transformation using immunofluorescence staining for E-cadherin, the expression of which is its cardinal feature (Cronan et al., 2016). All 8 eggs had E-cadherin staining, confirming that they had undergone epithelioid transformation as exemplified by Figure 2B; Video S2.

In mammals, *S. mansoni* eggs invoke macrophage-rich granulomas with very few neutrophils in contrast to *Schistosomajaponicum* eggs, which recruit both macrophages and neutrophils (Chensue et al., 1995; Moore et al., 1977; Swartz et al., 2006; Von Lichtenberg et al., 1973). Likewise, we found that in the zebrafish, granulomas forming to *S. mansoni* eggs contained very few neutrophils (Figures 2C and 2D). In contrast, similarly sized *Mycobacterium marinum* granulomas all contained neutrophils as expected (Figures 2C and 2D) (Yang et al., 2012). This pattern was established at the onset of egg implantation. Macrophages but not neutrophils were recruited at 6 h post-implantation (hpi), whereas the Gram-negative bacterium *Pseudomonas aeruginosa* recruited both types of cells, as expected (Figure 2E) (Yang et al., 2012). The lack of neutrophil recruitment has been attributed to the egg-secreted, interleukin-8-neutralizing *S. mansoni* chemokine-binding protein (smCKBP), more commonly known as alpha-1 (Smith et al., 2005). Accordingly, the injection of SEA recruited macrophages but not neutrophils, in contrast to *P. aeruginosa*, which recruited both (Figures 2F and 2G).

Next, we asked if the miracidium could survive within an epithelioid granuloma. We imaged individual eggs containing mature miracidia within organized granulomas at 5 dpi and found that they were still alive; the miracidium could be seen moving within the eggshell (Figure S3A; Video S3). E-cadherin staining immediately after imaging confirmed that the granuloma macrophages had indeed undergone epithelioid transformation (Figure S3B). We also saw that in those cases where the eggshell had ruptured either during or after implantation, macrophages had entered into the eggshell and destroyed the miracidium (Figure S3C; Video S3). These findings were consistent with those in mammals showing that the intact eggshell protects the miracidium against destruction by host macrophages (Bunnag et al., 1986; Hutchison, 1928; Von Lichtenberg et al., 1973). Further confirming this, miracidia implanted after collection from hatched eggs rapidly recruited macrophages that destroyed them (Figure S3D).

**Figure 3:**
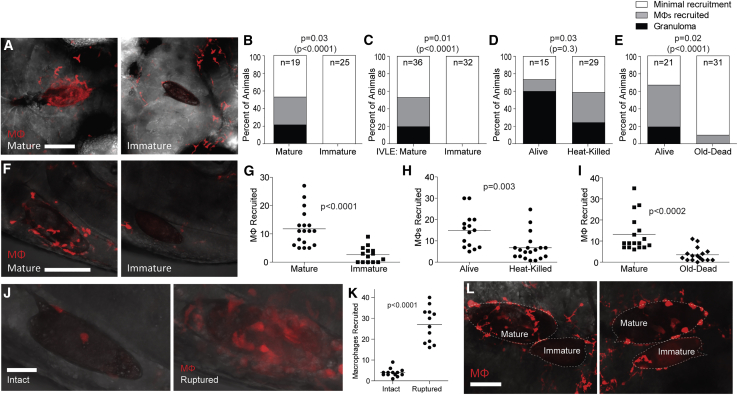
Immature Eggs Do Not Induce Macrophage Recruitment or Granuloma Formation (A–E) Granuloma formation and macrophage recruitment at 5 dpi comparing mature eggs with (A and B) immature eggs, (C) immature *in-vitro*-laid eggs (IVLE), (D) heat-killed eggs, or (E) old dead eggs. Representative images in (A), scale bar 100 μm. (B–E) Percent of animals with different levels of macrophage recruitment to the egg. (F–I) Macrophages recruited to mature eggs at 3 hpi compared with (F and G) immature eggs, (H) heat-killed eggs, and (I) old dead eggs. Representative images in (F), scale bar, 100 μm. (G–I) Quantification of macrophages recruited. (J) Confocal images showing macrophage recruitment to intact and mechanically ruptured immature eggs 6 hpi. Scale bar, 25 μm. (K) Quantification of macrophage recruitment to intact and ruptured immature eggs 6 hpi. (L) Confocal images of macrophage recruitment 5 dpi to co-implanted mature and immature eggs into the same HBV of two different larvae. Enumeration of recruited macrophages showed 19 and 2 macrophages recruited respectively to the mature and immature egg (left panel), and 23 and 6 macrophages recruited respectively to the mature and immature egg (right panel). Scale bar, 50 μm. (G–I) Horizontal bars, mean values. Statistics, (B–E) Fisher’s exact test comparing the proportion of eggs that induced granuloma formation (black bars), or granuloma formation with macrophage recruitment (black and gray bars combined, in parentheses); (G–I and K) Student’s t test. (B–E) n, number of animals. All experiments performed once, except for (F, G, J, and K), which are representative of two experiments. Also see Figure S4; Video S3.

Video S3. The Parasite Can Withstand Granuloma Formation if the Eggshell Is Intact, Related to Figures 2 and 3

In sum, we found that the key features of early mammalian responses to *S. mansoni* eggs are replicated in the zebrafish: selective macrophage recruitment to form bona fide epithelioid granulomas within days, which formed in the sole context of innate immunity. Our findings highlight that the miracidium tolerates granuloma formation as long as the eggshell is intact, a critical aspect of the *Schistosoma* life cycle that depends on granulomas to enhance egg extrusion from the host. These granulomas most closely resemble intestinal granulomas in mice, which comprise mostly macrophages with fewer lymphocytes and eosinophils (Weinstock and Boros, 1983).

### Immature *S. mansoni* Eggs Do Not Induce Macrophage Recruitment or Granuloma Formation

The egg matures 6 days after it is fertilized at which point it begins to secrete antigens (Ashton et al., 2001; Jurberg et al., 2009; Mann et al., 2011; Michaels and Prata, 1968). Accordingly, only viable mature eggs are found to induce granulomas (Jurberg et al., 2009; Von Lichtenberg et al., 1973). We sorted immature and mature eggs based on their size and appearance (Figure S4A) (Jurberg et al., 2009). None of the immature eggs had reached maturity by 5 dpi, and importantly all invoked only minimal macrophage recruitment (Figures 3A and 3B). To corroborate this result, we implanted *in-vitro*-laid eggs at 2 and 6 days post-fertilization in which the developmental stages were synchronized so that the 2-day eggs were immature and the 6-day eggs mature (Figure S4B). Again, the majority of the 6-day-old mature eggs induced macrophage recruitment, including granuloma formation, whereas the 2-day eggs elicited only minimal macrophage recruitment (Figure 3C). These results were consistent with antigens secreted from the mature egg being the trigger for granuloma formation (Ashton et al., 2001; Boros and Warren, 1970; Chiu and Chensue, 2002). To test this, we asked if dead eggs elicited a macrophage response. Freshly heat-killed eggs produced fewer granulomas than live eggs (Figure 3D). This finding is consistent with prior observations that some egg antigens are heat stable and that heat-killed eggs retain a thin layer of antigens which can induce granulomas, albeit less than living eggs (Freedman and Ottesen, 1988; Klaver et al., 2015; Von Lichtenberg, 1964). Accordingly, eggs stored at 4°C for 12 months so as to potentially inactivate all antigens (old dead eggs) did not induce granulomas, and only a minority (10%) recruited any macrophages at all (Figure 3E).

We next asked if immature and dead eggs, although failing to form granulomas, could still induce early transient macrophage recruitment. At 6 h post-implantation, immature, heat-killed, and old dead eggs all recruited fewer macrophages than live mature eggs (Figures 3F–3I). These findings suggested that mature egg antigens enhance macrophage recruitment from the earliest stages, and subsequently activate the recruited macrophages to form the granuloma.

Finally, we found that if we ruptured immature eggs prior to implantation, they rapidly recruited macrophages (Figures 3J and 3K). Similar to the case with ruptured mature eggs, these macrophages entered the ruptured immature egg and killed the embryo (Figure 3J and data not shown). Together these results suggest that while the exposed embryo and fully mature miracidium elicit macrophage recruitment similarly, the intact egg at the two stages is fundamentally different in its ability to recruit macrophages, the initial step that is required for granuloma formation.

To ask if egg antigen secretion was also required for the subsequent steps of macrophage aggregation into granulomas, we implanted an immature egg together with a mature egg in each animal. If mature egg antigens were required only to recruit macrophages to the egg, then the presence of the mature egg should recruit macrophages to the vicinity of the immature egg, allowing granulomas to form. In both instances, macrophages were recruited to and settled on the mature egg, with hardly any on the adjacent immature egg (Figure 3L). Thus, macrophage recruitment in response to the presence of egg antigens in the vicinity of the immature egg is not sufficient to induce macrophage adherence and granuloma formation. Rather, egg-intrinsic antigen is required for both macrophage recruitment and adherence to the egg with subsequent granuloma formation.

### The Immature *Schistosoma* Egg Evades Foreign Body Granuloma Formation

**Figure 4:**
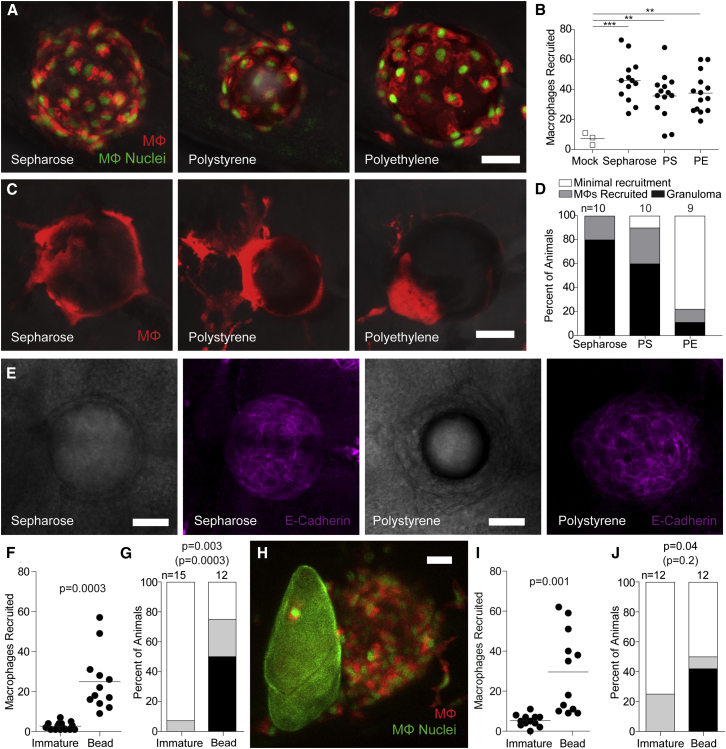
Chemically Inert Beads Induce Epithelioid Granulomas (A) Representative confocal images of macrophages recruited 6 hpi of sepharose, polystyrene, or polyethylene microspheres into the HBV of transgenic zebrafish larvae carrying red-fluorescent macrophages with green nuclei. (B) Enumeration of macrophages recruited to these microspheres in multiple animals. (C) Representative confocal images of granulomas formed around the three types of microspheres 5 dpi into the HBV of transgenic larvae carrying the transgene for red-fluorescent macrophages (without green nuclei). (D) Stages of macrophage recruitment to microspheres 5 dpi into HBV of multiple larvae. (E) Brightfield (panels 1 and 3) and fluorescence confocal (panels 2 and 4) microscopy of sepharose and polystyrene bead granulomas following immunofluorescence staining with the E-cadherin antibody. (F and G) Macrophage recruitment to immature eggs or microspheres implanted into the HBV at 6 hpi (F) and 5 dpi (G). (H–J) Macrophage recruitment following co-implantation of an immature egg and a polystyrene microsphere into the HBV of larvae transgenic for red-fluorescent macrophages with green nuclei. (H) Representative confocal image of an immature egg next to a microsphere. (I and J) Quantification of macrophage recruitment at 6 hpi (I) and at 5 dpi (J). Scale bars, 25 μm. (B, F, and I) Horizontal bars, means. (D, G, and J) n, number of animals. Statistics, one-way ANOVA (B), unpaired (F), and paired (I) Student’s t tests and Fisher’s exact test comparing granulomas (black bars) or granuloma formation with macrophage recruitment (black and gray bars, in parentheses) (G–J) Experiments in (E) and (F–J) were performed once. (A–D) are representative of three experiments. Also see Table S2.

Our findings were consistent with macrophage recruitment occurring only in response to antigens secreted from the mature egg rather than to the eggshell itself. Granulomas form in response to inert foreign bodies (Pagán and Ramakrishnan, 2018), so why would the eggshell not induce a foreign body granuloma? We considered three possibilities. First, that it was too small to invoke a foreign body response; this seemed unlikely as very small inert particles, e.g., a tiny thorn, can provoke a robust foreign body response (Pagán and Ramakrishnan, 2018). Second, that the mechanisms to form foreign body granulomas were not yet operant in the developing zebrafish larvae; this too seemed unlikely given that the foreign body granuloma response is evolutionarily ancient, and epithelioid granulomas form in response to foreign bodies in invertebrates (Pagán and Ramakrishnan, 2018). Third, that the immature schistosome egg has specific mechanisms to evade foreign body granuloma formation. To distinguish between these possibilities, we implanted beads of three different chemically inert materials of the same size as the schistosome egg (Table S2). We chose sepharose, which is hydrophilic, and polystyrene and polyethylene, which are hydrophobic. All recruited macrophages within 6 h (Figures 4A and 4B). By 5 days, epithelioid granulomas had surrounded most of the sepharose and polystyrene beads (Figures 4C–4E). The polyethylene beads were less granuloma inciting, with only 11% inducing bona fide granulomas, and most of the remaining beads failing to retain recruited macrophages (Figures 4C and 4D). However, even this weaker response was more robust than that of the immature eggs, which did not even transiently recruit macrophages. We confirmed these findings with a head-on comparison of macrophage recruitment and granuloma formation in response to immature eggs or similarly sized polystyrene beads in the same experiment (Table S2). Again, the polystyrene beads recruited macrophages by 6 h and formed granulomas by 5 days, whereas the immature eggs did neither (Figures 4F and 4G). This result suggested that the immature egg specifically avoids being recognized as a foreign body. This could be because the immature egg secretes a specific product to inhibit macrophage recruitment, or that the eggshell is immunologically inert. To distinguish between these possibilities, we implanted an immature egg and a polystyrene bead adjacent to each other in the same animal. In every case, at 6 h, macrophages were recruited only to the bead and not to the egg (Figures 4H and 4I). By 5 days post-implantation, granulomas had formed around the beads but none of the immature eggs (Figure 4J). These results support the idea that the eggshell evolved to be immunologically inert so as to evade the ubiquitous foreign body granulomatous response.

### Only Mature Eggs Translocate into the Intestinal Lumen of *S. mansoni*-Infected Mice and Humans

**Figure 5:**
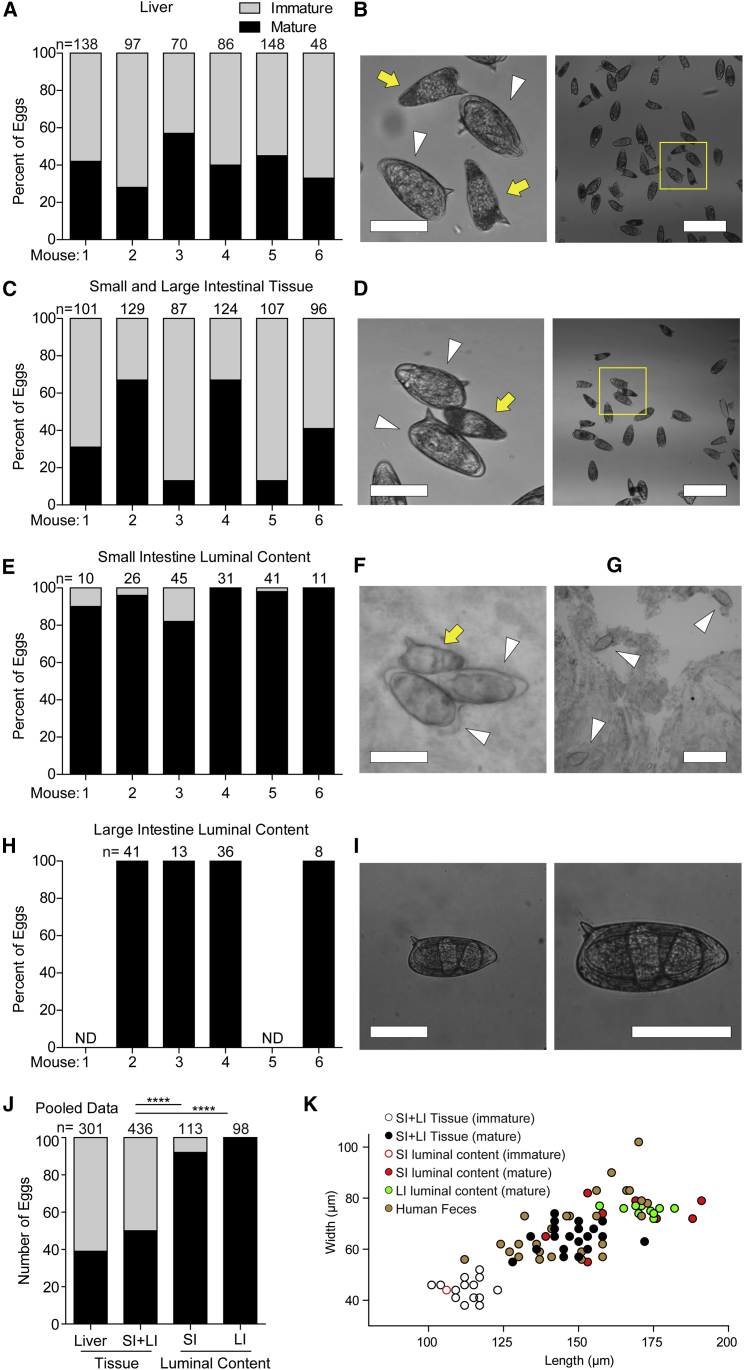
Mature Eggs Translocate into the Lumen of the Intestines (A–I) Quantification (A, C, E, and H) and representative brightfield images (B, D, F, G, and I) of mature and immature eggs found in the liver (A and B), small and large intestinal wall tissue and vasculature (C and D), small intestinal luminal content (E–G), and large intestinal luminal content (H and I) for six individual *S. mansoni*-infected mice. (B and D) Representative images with image (left) showing immature (yellow arrow) and mature (white arrowhead) magnified from yellow square in wide-field image (right). (F and G) Images of eggs from the lumen of the small intestine, showing two mature eggs in contact with one immature egg (F), and a wide-field image showing three mature eggs (G). (I) Representative image of an egg recovered from feces at low resolution (left) and higher resolution with developed miracidia visible (right). (J) Pooled data for mice 2, 3, 4, and 6 from (A, C, E, and H). SI, small intestine; LI, large intestine. (K) Dimensions of eggs from this experiment that were classified as immature or mature (open or closed circles, respectively) plotted with eggs shed in the feces of *S. mansoni*-infected humans (Martinez, 1916). All scale bars are 100 μm except for (G) and the right panels of (B and D), which are 300 μm. ND, not determined. Statistics, Fisher’s exact test. Also see Figure S5.

The observation that immature eggs, unlike mature eggs, are immunologically silent, led us to hypothesize, as Ashton et al. did (Ashton et al., 2001), that timing granuloma formation to egg maturation prevents the expulsion of immature eggs while they are still dependent on the absorption of nutrients from the host for development. Moreover, only a mature miracidium can survive in the aquatic environment and invade its snail host. If our hypothesis were true, we would expect to find in *S. mansoni*-infected mice, an enrichment of mature eggs in the intestinal lumen as compared with intestinal wall and liver. To test this prediction, mice were naturally infected with *S. mansoni* by cutaneous exposure to cercaria, and at 6 weeks post-infection, eggs were analyzed from liver, intestinal tissue, and small and large intestinal luminal content (feces). We quantified and categorized eggs as mature or immature by size and morphology (Jurberg et al., 2009) (Figures S4 and S5). As a test of our scoring accuracy, we then measured the size of the eggs and confirmed that our visual inspection had correctly separated the immature and mature eggs (Figure S5). We next assessed the distribution of mature and immature eggs for each collection site in each mouse. We found that while the liver and the intestinal tissue contained roughly equal proportions of both immature and mature eggs (Figures 5A–5D), hardly any immature eggs were found in the small intestinal lumen (6% average for all six animals; Figure 5E). Moreover, only 2 out of 11 immature eggs were at the very early stage of development, with the remaining ones nearing maturity (Jurberg et al., 2009) (Figures 5E–5G). All eggs scored from the lumen of the large intestines (feces) were morphologically mature and contained fully mature miracidia (Figures 5H and 5I). Statistical analysis of the pooled data from four mice confirmed an enrichment of mature eggs in the lumen of the small and large intestines (Figure 5J). These results confirmed that virtually all eggs shed by infected mice are mature.

Do humans also shed only mature eggs? We were unable to find a direct answer to this question in the literature. However, we found a paper that had assessed the length and width of 30 eggs shed in the feces of *S. mansoni*-infected humans (Martinez, 1916). Because we had found that immature and mature eggs differ in size with immature eggs being much smaller (Figures S4 and S5) (Ashton et al., 2001), we were in a position to determine if the eggs shed by humans were mature or immature. We plotted the sizes of the eggs shed in human feces alongside the eggs from the mouse intestinal wall and lumen and found that all of the human eggs were in the mature egg size range (Figure 5K). Thus, humans also shed only mature eggs.

These results support the hypothesis that the timing of granuloma formation and subsequent egg expulsion is modulated so as to prevent premature expulsion of immature eggs, which would be terminal for the parasite.

## Discussion

Research on *S. mansoni* granulomas has focused mainly on the organ-damaging fibrosis that ensues from granulomas forming around tissue-lodged eggs (Colley and Secor, 2014). Yet most *S. mansoni*-infected individuals are either asymptomatic or only mildly symptomatic (Hams et al., 2013), possibly because their granulomatous response is more tempered. These individuals shed parasite eggs, highlighting that disease per se does not benefit the parasite’s evolutionary survival. Rather, as in the case with many infectious diseases, human disease represents collateral damage stemming from the host-pathogen interaction, harming the host with little benefit to the pathogen (Relman et al., 2020). On the other hand, early granuloma formation in appropriate anatomical locations is thought to benefit both host and parasite for the same reason, expelling the parasite egg from the human host so as to enable it to continue its life cycle in its intermediate snail host (Dunne et al., 1983; Hams et al., 2013). While this idea is appreciated, it has been difficult to study extensively because of experimental limitations. Early or asymptomatic human infection seldom presents itself for study, and existing animal models are less suitable for the study of early granuloma-associated pathology.

This work explores the earliest steps of *Schistosoma* granuloma formation that have not been captured in existing animal models. We show that as is the case with mycobacterial granulomas, bona fide epithelioid granulomas form in response to the *Schistosoma* egg in the sole context of innate immunity (Cronan et al., 2016; Davis et al., 2002). This should not be surprising given that epithelioid granulomas form in multiple invertebrate species in response to retained foreign bodies or even their own dead eggs (Pagán and Ramakrishnan, 2018). Yet, there has been a limited appreciation that adaptive immunity is dispensable for the formation of such an organized structure in the context of infectious granulomas, and indeed, the emphasis of schistosomiasis research on the late-stage granuloma has caused the focus to be on how the granuloma is modulated by adaptive immunity to become pathogenic (Hams et al., 2013; Pagán and Ramakrishnan, 2018). Given that *Schistosoma* eggs begin to be shed into the feces within days following maturation (deWalick et al., 2012), egg shedding must occur even in the absence of adaptive immunity and is likely promoted by these innate epithelioid granulomas. Our finding of the rapid epithelioid transformation of the granuloma also has relevance for granuloma-induced transmission later in infection when adaptive immunity is operant. Moreover, intestinal granulomas, the ones that extrude the eggs, are smaller than those in the liver, with a paucity of the lymphocytes and eosinophils that characterize liver granulomas (Weinstock and Boros, 1983). The rapid epithelioid transformation of the *Schistosoma* granuloma may help it to extrude the eggs more efficiently.

We have also gained understanding of the mechanics of early granuloma formation. Broadly speaking, granuloma formation in response to the mature egg proceeds in two discrete steps. In the first step, macrophages are attracted to secreted parasite antigens, and upon contact with the egg, appear to gain a chemotactic activity that outstrips that of the egg. This results in the subsequent macrophages being recruited to the existing macrophages forming a tight, aggregate that then pulls itself together to encapsulate the egg. It is noteworthy that epithelioid transformation precedes the complete covering of the egg, highlighting that this specialized macrophage transformation (Pagán and Ramakrishnan, 2018) constitutes an early response.

While these new details on how granulomas form around mature eggs are thought provoking, more striking is the lack of even minimal macrophage recruitment to the immature egg. Given that like-sized beads recruit macrophages robustly and induce epithelioid granulomas, this finding reveals further nuance to the exploitation of the granuloma by the parasite. Not only must the parasite induce granuloma formation through secretion of antigens, but it must also prevent the granuloma from forming too soon. The egg is laid into the bloodstream, and needs to extravasate to reach the gut wall (deWalick et al., 2012). This process takes at least 6 days, perfectly synchronized with the time it takes for the miracidium to mature (Michaels and Prata, 1968; Pellegrino et al., 1962). The mature egg, now in the intestinal wall, will induce the granulomas that promote its extrusion. Premature granuloma formation could be detrimental to the parasite for two reasons. It could encumber its passage to the intestinal wall. Conversely, premature extrusion would remove the egg from the human tissue environment that is essential for its maturation (Ashton et al., 2001).

Prior work has noted that the granuloma-inducing secreted *Schistosoma* antigens are secreted from the egg, rather than being incorporated into the eggshell, and that secretion occurs only after egg maturation (Ashton et al., 2001; Schwartz and Fallon, 2018). This work adds the insight that the immunologically inert nature of the eggshell is a requisite counterpart of the *Schistosoma* transmission strategy. Our ability to directly compare granuloma formation around eggs and beads has been key to this insight. It will be interesting to determine how the eggshell remains immunologically inert in the context of adaptive immunity, particularly because eggshell proteins induce antibodies in humans (Dewalick et al., 2011; deWalick et al., 2012). Foreign body granuloma formation is a major complication of implanted devices (Pagán and Ramakrishnan, 2018). Identifying the chemical basis of the granuloma-silencing mechanism of the eggshell may have therapeutic implications in the design of inert materials for medical implants that prevent foreign body granulomas.

## STAR★Methods

### Key Resources Table

**Table:** 

REAGENT or RESOURCE	SOURCE	IDENTIFIER
**Antibodies**		

Rabbit polyclonal antibody against L-plastin	Abcam	ab210099
Alexa Fluor 555 Goat anti-Rabbit IgG (H + L) antibody	ThermoFisher	A-21428; RRID: AB_141784
Mouse anti-E-cadherin antibody, clone 36	Becton Dickinson	CAT# 610181; RRID: AB_397580
Alexa Fluor 647 Goat Anti-Mouse IgG (H + L) antibody	ThermoFisher	A-21236; RRID: AB_2535805

**Bacterial and Virus Strains**		

*Mycobacterium marinum* M strain/pMSP12:EBFP2	Takaki et al., 2013	KT30
*Pseudomonas aeruginosa* MPAO1 strain	Gift from Gordon Dougan, University of Cambridge	MPAO1

**Biological Samples**		

*Schistosoma mansoni* eggs Puerto Rican strain	Gabriel Rinaldi, this study	N/A
Soluble Egg Antigens	Gabriele Schramm, this study	N/A

**Chemicals, Peptides, and Recombinant Proteins**		

Sepharose agarose microspheres	Sigma	CAT#C9142
Polyethylene microspheres (CPMS-0.96 63-75 μm)	Cospheric	Item#CPMS-63-75um
Polyethylene microspheres (CPMS-0.96 27-32 μm)	Cospheric	Item#CPMS-27-32um
Polystyrene microspheres (45 μm)	Generon	CAT#07314-5
Tricaine	Sigma	SKU# A5040
Instant Ocean	Spectrum Brands	N/A
PTU (1-phenyl-2-thiourea)	Sigma-Aldrich	SKU# P7629
Normal goat serum (10%)	ThermoFisher	CAT# 50197Z
Triton X-100	Sigma-Aldrich	SKU# T8787
Bovine Serum Albumin (BSA)	Sigma-Aldrich	SKU# A7906
Fetal Bovine Serum (FBS)	ThermoFisher	CAT# 10082147
Clostridial collagenase	Sigma-Aldrich	SKU# C5138
HEPES (1M)	ThermoFisher	CAT# 15630080
DMEM	ThermoFisher	CAT# 11965092
Modified Basch’s Medium	Mann et al., 2010	N/A
Dimethyl sulfoxide (DMSO)	Fisher Scientific	CAT# BP231-100
Low Melting Point (LMP) Agarose	Invitrogen	CAT# 16520-100
(Hydroxypropyl)methyl cellulose	Sigma	SKU# H7509-25G
Micro-BCA assay	Pierce Biotech Inc.	CAT# 23225
Optical bottom plates	MatTek Corporation	P06G-1.5-20-F
Antibiotic-Antimycotic (100X)	ThermoFisher	CAT# 15240062
Euthatal Solution for Injection (200 mg/ml)	Dopharma B.V.	N/A
Hygromycin B	Mediatech	30-240-CR
7H9 Middlebrook broth base	Difco	CAT# 271310
Acrodisc 5 μm syringe filter	VWR	28144-095

**Experimental Models: Organisms/Strains**		

*Schistosoma mansoni* NMRI Puerto Rican strain	BEI Resources	https://www.beiresources.org/Catalog/BEIParasiticWorms/NR-21962.aspx
*Biomphalaria glabrata*	Wellcome Sanger Institute	N/A
HsdOla:TO female mice	Envigo, UK	https://www.envigo.com/model/hsdola-to
Zebrafish (*Danio rerio*): wild type AB strain	Zebrafish International Resource Center	ZDB-GENO-960809-7
Zebrafish: *Tg(mpeg1:Brainbow)*^*w201*^	Pagán et al., 2015	ZDB-FISH-151204-7
Zebrafish: *Tg(lyz:EGFP)*^*nz117*^	Hall et al., 2007	ZDB-TGCONSTRCT-071109-2
Zebrafish: *Tg(mfap4:nlsVenus-2A-tdTomato-CAAX)*	(A. Pagán, this study)	N/A

**Software and Algorithms**		

NIS-Elements 4 (Version 5.21.01)	Nikon	https://www.microscope.healthcare.nikon.com/products/software/nis-elements/nis-elements-advanced-research
Imaris X64	Bitplane	https://imaris.oxinst.com/
Prism 5.01	GraphPad	https://www.graphpad.com/
Illustrator CS5	Adobe	https://www.adobe.com/products/illustrator.html?promoid=PGRQQLFS&mv=other

**Other**		

Values for the human data on schistosomiasis eggs	Martinez, 1916	n/a

### Resource Availability

#### Lead Contact

Further information and requests for resources and reagents should be directed to and will be fulfilled by the Lead Contact, Lalita Ramakrishnan (lr404@cam.ac.uk).

#### Materials Availability

All unique and stable reagents generated in this study are available from the Lead Contact. Some restrictions may apply.

#### Data and Code Availability

This study did not generate any unique datasets or code.

### Experimental Model and Subject Details

#### Ethics Statement

All animal experiments were conducted in compliance with guidelines from the UK Home Office and approved by the Wellcome Sanger Institute (WSI) Animal Welfare and Ethical Review Body (AWERB).

#### Zebrafish Husbandry

All zebrafish lines were maintained on a recirculating aquaculture system with a 14 hour light - 10 hour dark cycle. Fish were fed dry food and brine shrimp twice a day. Zebrafish embryos were housed in fish water (reverse osmosis water containing 0.18 g/l Instant Ocean) at 28.5°C. Embryos were maintained in 0.25 μg/ml methylene blue from collection to 1 day post-fertilization (dpf). At 24 h post-fertilization 0.003% PTU (1-phenyl-2-thiourea, Sigma) was added to prevent pigmentation.

#### Fish Lines

Experiments requiring larvae with red-fluorescent macrophages were performed using Tg(mpeg1:Brainbow)^w201^ (Pagán et al., 2015). For experiments requiring analysis of neutrophils, Tg(lyz:EGFP)^nz117^ (Hall et al., 2007) were crossed with Tg(mpeg1:Brainbow)^w201^ (Pagán et al., 2015) to produce larvae with green neutrophils and red macrophages. Experiments assessing early macrophage recruitment in response to beads or ruptured immature eggs utilized Tg(mfap4:nlsVenus-2A-tdTomato-CAAX)(A.J.P., unpublished data). All zebrafish lines were produced in an AB background, with the exception of Tg(mfap4:nlsVenus-2A-tdTomato-CAAX) which utilized a mixed AB/TLF background.

#### Mouse Model

Mouse experimental infections and regulated procedures were conducted under Home Office Project License No. P77E8A062 held by G. Rinaldi. Outbred HsdOla:TO female mice were housed individually in ventilated cages (Tecniplast, IsoCage N -Biocontainment Systems) and maintained on individual air handling units at 19-23°C and 45–65% humidity. Animals were given access to food and water ad libitum, maintained on a 12-hour light/dark cycle.

#### Snail Husbandry

*Biomphalaria glabrata* snails (NMRI strain) were maintained and bred at the Wellcome Sanger Institute as described (Tucker et al., 2013). Briefly, snails were kept in aerated aquaria tanks, at 28°C, on a 12-h light/dark cycle, and were fed twice weekly with gel food and egg masses collected from the breeder tanks once a week. Snails were infected by exposure to 30 *S. mansoni* miracidia and then housed at 28°C protected from light.

#### Bacterial Strains

*Mycobacterium marinum* M strain/pMSP12:EBFP2 were prepared and described previously (Takaki et al., 2013). Briefly, *Mycobacterium marinum* M strain/pMSP12:EBFP2 was grown in 7H9 Middlebrook broth containing 50 mg/mL hygromycin B at 33°C without shaking for 1 week until reaching an optical density of 0.5-1.0 OD600. Bacteria were then passed through a 1 mL tuberculin syringe with a 27-gauge needle, and then passed through a 5 μm syringe filter. The resulting single-cell bacteria were aliquoted and stored at -80°C. *Pseudomonas aeruginosa* was cultured in LB medium overnight at 37°C and then aliquoted and stored at -80°C.

#### Schistosoma Strains

The complete life cycle of *Schistosoma mansoni* NMRI (Puerto Rican) strain is maintained at the WSI by breeding and infecting susceptible *Biomphalaria glabrata* snails, and mice.

### Method Details

#### Isolation and Manipulation of Schistosome Eggs

The complete life cycle of *Schistosoma mansoni* NMRI (Puerto Rican) strain is maintained at the WSI by breeding and infecting susceptible *Biomphalaria glabrata* snails, and mice. Schistosome eggs were harvested as previously described (Mann et al., 2010). Briefly, anesthetized Balb/c female mice were infected by tail submersion in water containing 250 *S. mansoni* cercariae collected from experimentally infected snails, and 6 weeks later euthanized by an overdose of Euthasol (sodium pentobarbital and sodium phenytoin, 40 mg per mouse) delivered by intraperitoneal injection. Mixed-sex adult worms were collected by portal perfusion, washed and maintained in culture for *in vitro* laid eggs (IVLE) collection (below). The mouse livers were removed after the portal perfusion, minced with a sterile razor blade in 1X PBS containing 200 U/ml penicillin, 200 μg/ml streptomycin and 500 ng/ml amphotericin B (i.e. 2% antibiotic-antimycotic - ThermoFisher Scientific), and incubated with 5% clostridial collagenase (Sigma) in 1X PBS with 2% antibiotic-antimycotic at 37°C with shaking for 16 h. The digested liver tissue mixed with the schistosoma eggs was washed three times with 1X PBS with 2% antibiotic-antimycotic by centrifugation at 400 g for 5 min at room temperature and serially filtered through a sterile 250 μm and 150 μm sieve. The eggs were then separated from the liver tissue by a sucrose-based Percoll gradient and washed three times as above. The eggs were kept at 37°C, 5% CO_2_ in DMEM supplemented with 10% FBS and 2% antibiotic-antimycotic. All the procedures were performed in sterile conditions inside a biological safety cabinet. For experiments comparing eggs from liver and gut, and from the luminal contents of the small and large intestines, liver, and gut eggs were isolated as above, but with serial passage through 300 μm and 200 μm filters (pluriSelect, 43-50300-01 and 43-50200-01). For isolation of eggs from the lumen of the small and large intestines, the luminal content was gently extracted by squeezing the intestines, which contained both intestinal feces and translocated eggs.

*S. mansoni* IVLE were harvested as previously described (Mann et al., 2010; Rinaldi et al., 2012). Briefly, schistosome mixed-sex worms collected by portal perfusion were washed with sterile 1X PBS and 2% antibiotic-antimycotic, placed in 6-well plates and cultured in modified Basch’s medium (Mann et al., 2010) at 37°C, 5% CO_2_. Two days later, the eggs laid *in vitro* by the cultured worm pairs were collected from the bottom of the well. For experiments using immature IVLE, eggs were implanted into zebrafish larvae soon after collection, and for experiments using mature IVLE, eggs were cultured in modified Basch’s medium at 37°C, 5% CO_2_ for 6 days before being implanted into zebrafish larvae. For experiments using heat-killed eggs, the eggs were killed at 90°C for 15 min and incubated in 1 mL of modified Basch’s medium for 3 days to wash away residual egg antigens. Old dead eggs were created by stored at 4°C for >12 months and were verified as unviable based on lack of miracidial movement and hatching. For experiments using ruptured immature eggs, the CAIN was used to apply downwards pressure in combination with a sideways motion over the glass slide.

#### Implantation of Schistosoma Eggs

Capillary-Assisted Implantation Needles (CAIN) were created by pulling borosilicate thin wall with filament capillaries (GC100TF-10, Harvard Instruments) using a micropipette puller (Sutter Instruments, P-2000) with the following settings: Heat = 350, FIL = 4, VEL = 50, DEL = 225, PUL = 150. The tips of pulled needles were opened with jeweler’s forceps and then double-beveled using a MicroForge-Grinding Center (MFG-5, Harvard Instruments). Micromanipulation was achieved using a 3-axis micromanipulator (Narishige, M-152) with pressure control using a FemtoJet Express microinjection unit (Eppendorf). The VAMP (Vacuum-Assisted MicroProbe) was previously described (Takaki et al., 2013).

Larval zebrafish were anesthetized and implanted at 30 hpf in 0.252 g/L tricaine (Sigma, A5040) in a modified Schistosomula Wash medium (500 ml DMEM, 5 ml 1M HEPES and 2% antibiotic-antimycotic) to prevent egg hatching during implantation. Anesthetized larvae were grasped using the VAMP and an incision was made in the forebrain region using the CAIN. After making an incision, a single schistosome egg was picked up using the capillary action of the CAIN, and passed through the incision and deposited into the hindbrain ventricle (Video S1).

#### Implantation of Beads

Zebrafish larvae were implanted with Sepharose (Sigma, C9142), polyethylene (Cospheric, CPMS-0.96 63-75um and CPMS-0.96 27-32um), and polystyrene (Generon, 07314-5) microspheres in fish water containing 0.252 g/L tricaine (Sigma, A5040) using the same technique as with schistosome egg implantations. Bead volumes calculated using the median radius (1/2 diameter) and formula for the volume of a sphere (v=4/3πr^3^). Egg volumes determined by 3D confocal microscopy.

#### Hindbrain Ventricle Microinjections

Hindbrain ventricle injection of bacteria and soluble reagents were performed under anesthesia with 0.252 g/L tricaine (Sigma, A5040) using a microinjection needle supplied to a FemtoJet Express microinjection unit (Eppendorf), with larval manipulation performed using the VAMP (Takaki et al., 2013) and (Video online).

#### Bacterial Infections

Zebrafish larvae were infected with 20 CFU of *Mycobacterium marinum* M strain (ATCC #BAA-535) constitutively expressing EBFP2 (strain KT30) (Takaki et al., 2013), or 200 CFU of *Pseudomonas aeruginosa* (strain MPAO1, courtesy of Professor Gordon Dougan). All bacterial procedures were performed using dedicated equipment separate from *Schistosoma* procedures, and disinfected with 70% EtOH after use.

#### Soluble Egg Antigens (SEA)

SEA was prepared by Dr Gabriele Schramm. Briefly, eggs were isolated from *S. mansoni*-infected hamsters as previously described (Schramm et al., 2018), and then homogenized in PBS, pH 7.5, using a sterile glass homogenizer. The homogenate was then centrifuged at 21 krcf for 20 min. Supernatants were pooled and then dialyzed overnight in PBS using a 3.5 kDa molecular weight cutoff dialyzer. Sample was then centrifuged at 21 krcf for 20 min, and supernatant (SEA) was aliquoted and stored at -80°C. SEA was quantified for protein concentration using the Micro-BCA assay (Pierce, 23225), and quality controlled by SDS-PAGE and western blotting against the *S. mansoni* antigens, Omega-1, Alpha-1, and Kappa-5. Quality control for low LPS content was performed using the Chromo-LAL assay (Associate of Cape Cod, Inc., C0031-5). SEA was injected at 2 ng per hindbrain ventricle (1.5 nL injection of 1.4 mg/mL SEA).

#### Immunofluorescence Staining

Immunofluorescence was performed as previously described (Cronan et al., 2016). Briefly, zebrafish larvae were fixed in Dent’s fixative overnight at 4°C, rehydrated in PBS containing 0.5% tween 20, and then blocked for 1 h in PBDTxGs (PBS containing 1% BSA, 1% DMSO, 0.1% Triton X-100, 2% goat serum). Mouse anti-E-cadherin antibody, clone 36 (BD, 610181) was added at a 1/500 dilution followed by incubation overnight at 4°C. Larvae were washed in PBDTxGs and then Alexa Fluor 647 Goat Anti-Mouse IgG (H+L) antibody (ThermoFisher, A-21236) added at a 1/500 dilution followed by incubation overnight. Larvae were washed 5 times in PBDTxGs before analysis.

#### Analysis of Eggs from Liver, Gut, and Small and Large Intestines

All eggs were analyzed by microscopy and scored as immature or mature based morphological differences in size and shape as characterized by Jurberg (Jurberg et al., 2009). Eggs from the liver and gut were analyzed in 1x PBS. Eggs from the small intestine were imaged in a petri plate using a glass coverslip to create a thin section of sample to image through. Eggs from the large intestines were diluted in 2% methyl cellulose and spread thinly across a petri plate to dilute the fecal matter and create a thin section of sample for analysis.

#### Confocal Microscopy

Zebrafish were anesthetized in fish water containing tricaine and then and mounted onto optical bottom plates (Mat Tek Corporation, P06G-1.5-20-F) in 1% low melting point agarose (Invitrogen, 16520-100) as previously described (Takaki et al., 2013). Microscopy was performed using a Nikon A1 confocal laser scanning confocal microscopy with a 20x Plan Apo 0.75 NA objective and a Galvano scanner, acquiring 30-80 μm z-stacks with 2-3 μm z-step intervals. Timelapse microscopy was performed at physiological temperature using a heat chamber set to 28°C (Okolab) with an acquisition interval of 2.5-3 min. For multi-day timelapse imaging (Figures 2A and S2), zebrafish larvae were carefully removed using jeweler's forceps and returned to their standard housing (see husbandry) for imaging at later timepoints.

### Quantification and Statistical Analysis

#### Phagocyte Recruitment

For quantification of phagocyte recruitment, fluorescence confocal microscopy was performed, capturing z-stack images at the designated timepoint following implantation of eggs or beads, or the injection of soluble antigens or bacteria. Experimental groups were then blinded, and 3D rendering of confocal images were used to count the number of phagocytes in contact with the schistosome egg or bead, or the number of phagocytes within the hindbrain ventricle following injection of soluble antigens or bacteria.

#### Determination of Egg Volume

Schistosome eggs were stained with Coomassie InstantBlue dye (Sigma, ISB1L) and imaged by confocal microscopy with the 641 nm laser and CY5 HYQ filter, 590-650 nm excitation and 663-738-nm emission. Using Imaris X64 (Bitplane) 3D surface rendering of the eggs were then generated and used to calculate the egg volumes.

#### Macrophage Tracking

Time-lapse confocal images were used to generate 3D surface rendering of macrophages which were tracked over time using Imaris X64 (Bitplane).

#### Statistical Analysis

Statistical analyses were performed using Prism 5.01 (GraphPad Software), with each statistical test used specified in the corresponding figure legend. Post-test p values are as follows: ns, not significant; ^∗^ p < 0.05; ^∗∗^ p < 0.01; ^∗∗∗^ p < 0.001; ^∗∗∗∗^ p < 0.0001. Where the n value is given and not represented graphically in the figure, n represents the number of zebrafish used for each experimental group.

## Supplemental Information

Document S1. Figures S1–S5 and Tables S1 and S2

Document S2. Article plus Supplemental Information
