# Supplementary material for: Schistosoma mansoni Eggs Modulate the Timing of Granuloma Formation to Promote Transmission
Source: Cell Host Microbe. 2021 Jan 13;29(1):58–67.e5. doi: 10.1016/j.chom.2020.10.002 (PMC7815046; doi:10.1016/j.chom.2020.10.002)
Supplement: Document S1. Figures S1–S5 and Tables S1 and S2 [file mmc1.pdf]

Cell Host & Microbe, Volume *29*

Supplemental Information

Schistosoma mansoni Eggs Modulate the Timing of Granuloma Formation to Promote Transmission

Kevin K. Takaki, Gabriel Rinaldi, Matthew Berriman, Antonio J. Pagán, and Lalita Ramakrishnan

Cell Host & Microbe

Supplemental Information

Schistosoma mansoni eggs modulate the timing of granuloma formation to promote transmission

Kevin K. Takaki, Gabriel Rinaldi, Matthew Berriman,
Antonio J. Pagán, Lalita Ramakrishnan

SUPPLEMENTAL CONTENTS

Table S1. Prevalence of granuloma formation, related to Figure 1

Table S2. Sizes of implanted materials, related to Figure 4

Figure S1. Implantation of schistosome eggs into zebrafish larvae, related to Figure 1

Figure S2. Formation of the epithelioid granuloma, related to Figure 2

Figure S3. The eggshell protects the miracidium from being killed by host macrophages, related to Figure 2

Figure S4. Morphology and volume of mature and immature eggs, related to Figure 3

Figure S5. Dimensions and volume of mature and immature eggs , related to Figure 5

Supplementary Tables

Table S1. Prevalence of granuloma formation

Exp	Minimal Recruitment (%)	MΦs Recruited (%)	Granuloma (partial)(%)	Granuloma (complete)(%)	Granuloma (%)	n
1	7 (44%)	5 (31%)	3 (19%)	1 (6%)	4 (25%)	16
2	1 (20%)	2 (40%)	2 (40%)	0 (0%)	2 (40%)	5
3	8 (44%)	2 (11%)	7 (39%)	1 (6%)	8 (44%)	18
4	5 (25%)	6 (30%)	6 (30%)	3 (15%)	9 (45%)	20
5	15 (38%)	11 (28%)	10 (25%)	4 (10%)	14 (35%)	40
6	2 (25%)	5 (63%)	1 (13%)	0 (0%)	1 (13%)	8
7	2 (17%)	8 (67%)	2 (17%)	0 (0%)	2 (17%)	12
8	3 (43%)	4 (57%)	0 (0%)	0 (0%)	0 (0%)	7
Total	43	43	19%	6%	40	126
Mean %	32%	41%	23%	5%	28%	---

The number and rounded percentages of implanted eggs in each category of immune response as defined in **Figure 1D**, for each of 8 experiments. Each experiment constitutes a separate batch of eggs and a separate clutch of zebrafish larvae. Mean percent is the mean of the percent of eggs in each category for each individual experiment. Sample size, n, the total number of assessed eggs per experiment. Linked to Figure 1D and 1E.

Table S2. Sizes of implanted materials

**Implanted Material**	**Diameter (median, µm)**	**Volume (median, μm^3)**
Mature Schistosome egg	---	200,000
Immature Schistosome egg	---	60,000
Sepharose Agarose beads	65	146,346
Polystyrene beads	45	47,713
Polyethylene beads (large)	70	175,909

Linked to Figure 4.

Supplementary Figures

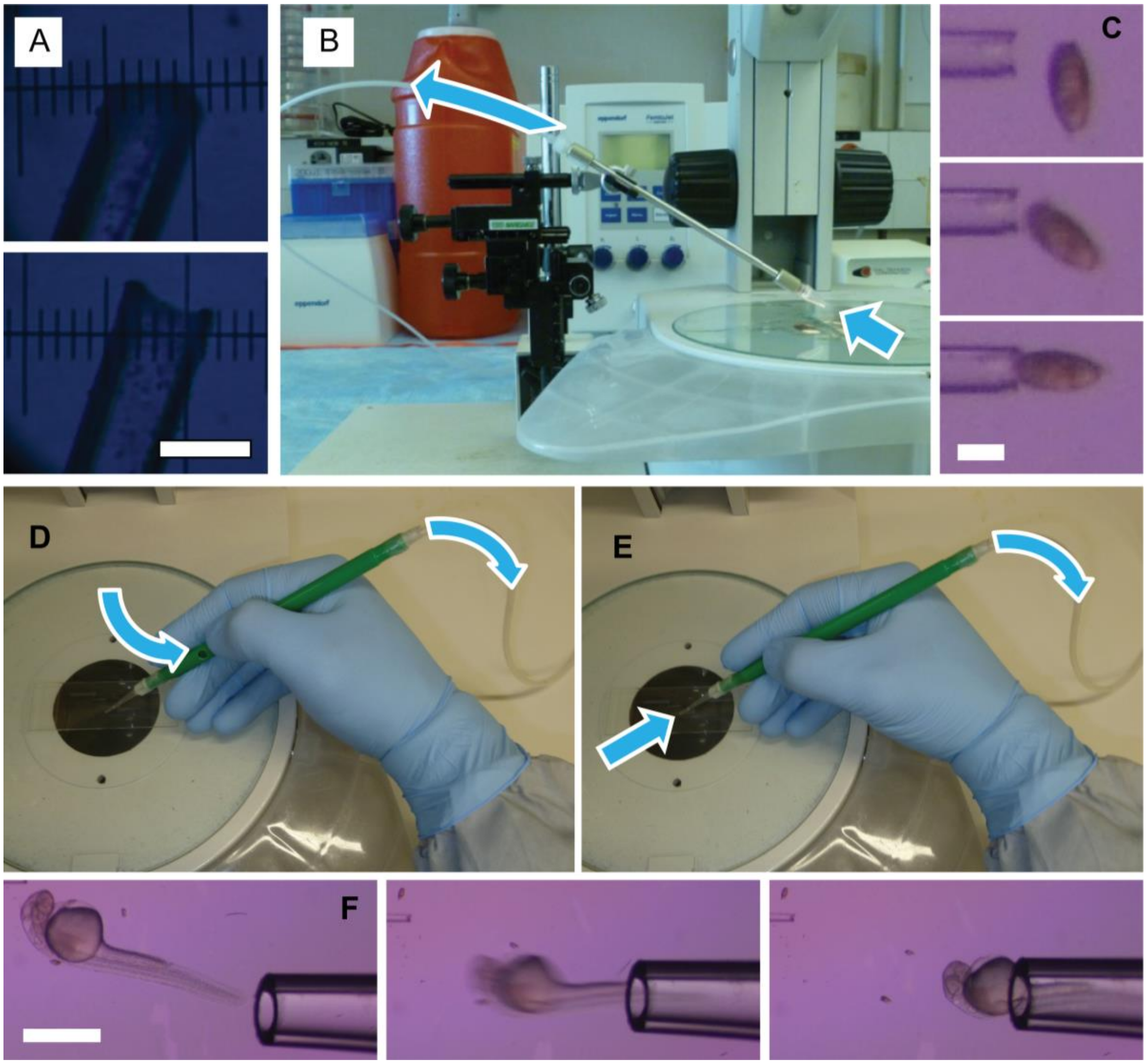

Figure S1. Implantation of schistosome eggs into zebrafish larvae
(**A-C**) Capillary-Assisted Implantation Needle (CAIN). (**A**) Side and front profile of CAIN showing double-beveled point. Scale bar 50 μm. (**B**) CAIN attached to micromanipulator for X,Y, and Z control, as used by left hand of operator. Arrows indicated upward flow of fluid during grasping of egg. (**C**) Function of CAIN demonstrated by grasping *S. mansoni* egg. Scale bar, 50 μm. (**D-F**) Vacuum-Assisted MicroProbe (VAMP). (**D**) Occlusion of thumb hole re-routes aspiration pressure to tip (**E**), allowing for grasping of the larvae (**F**). Scale bar, 1000 μm. VAMP as previously described (Takaki et al., 2013). Linked to Figure 1 and Video S1.

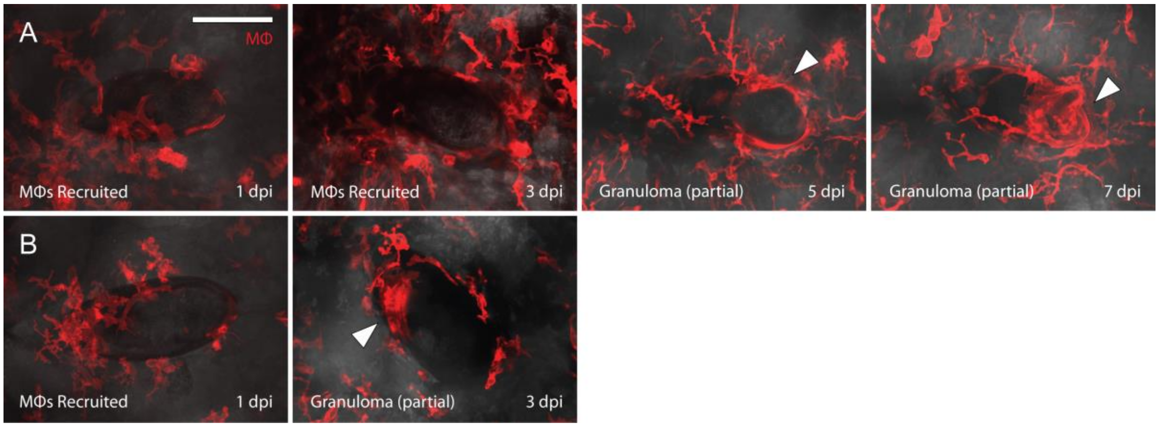

Figure S2. Formation of the epithelioid granuloma
Timelapse microscopy following the formation of the epithelioid granuloma (white arrowhead) from 1-7 dpi, imaged at 2 day intervals. Two examples shown, (**A**) and (**B**). Animal in (**B**) was not recovered after the 3 day time point. Scale bar, 100 µm. Linked to Figure 2A.

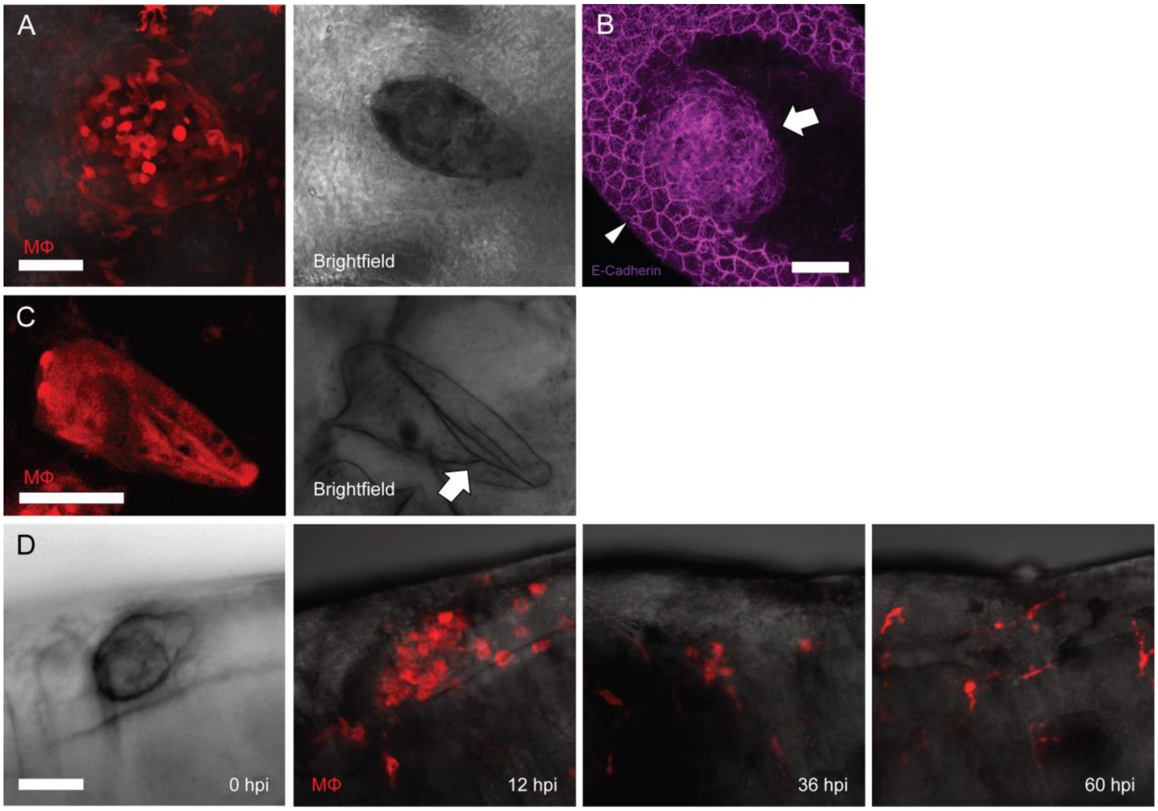

Figure S3. The eggshell protects the miracidium from being killed by host macrophages
(**A** and **B**) The parasite is alive within an epithelioid granuloma at 5 dpi. (**A**) Fluorescence and brightfield intravital microscopy. (**B**) Immunofluorescence staining with E-cadherin antibody. The outer-most stained structure is the epithelial lining of the hindbrain ventricle (arrowhead), and is not in contact with the epithelioid granuloma (arrow). (**C**) Fluorescence and brightfield microscopy of ruptured egg showing macrophage infiltration and the absence of an intact parasite. Arrow, rupture point of eggshell. (**D**) Representative brightfield and fluorescence timelapse microscopy of a miracidium following implantation into the HBV. (**A**-**C**) Representative of routinely observed miracidia alive within intact eggs within granulomas, and of occasionally ruptured eggs. (**D**) Representative of two experiments, each with a sample size of 10 animals. Scale bars, 50 µm. Linked to Figure 2 and Video S3.

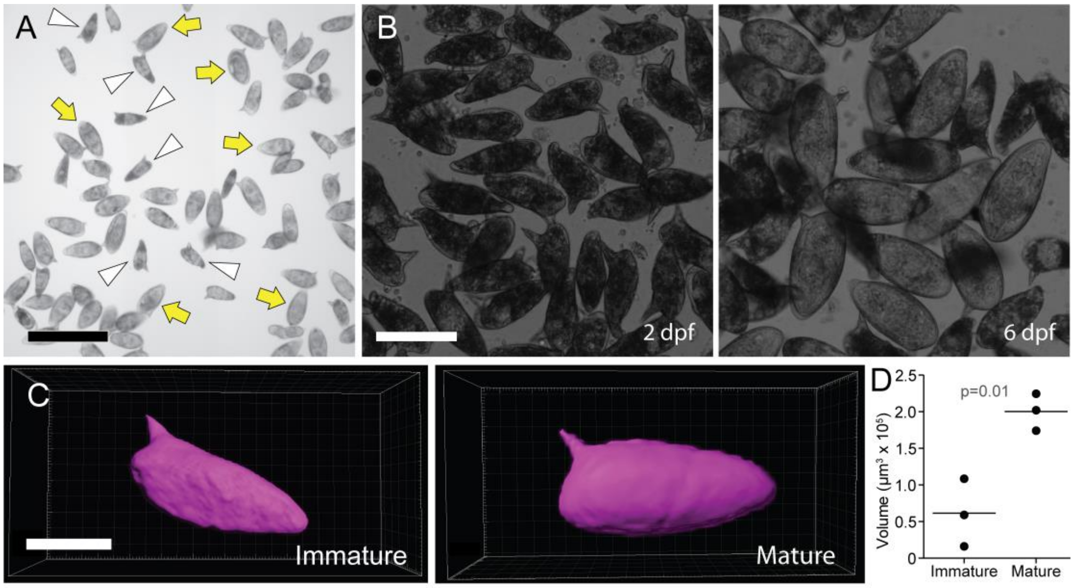

Figure S4. Morphology and volume of mature and immature eggs
(**A**) *S. mansoni* eggs isolated from mouse livers. Immature and mature eggs, arrowheads and arrows, respectively. Scale bar, 300 µm. (**B**) Immature IVLE at 2 days post-fertilization (dpf), and mature IVLE at 6 days post-incubation in nutrient medium at 37°C. Scale bar, 100 µm. (**C**) 3D rendering of Coomassie-stained eggs following confocal microscopy, and (**D**) volumetric analysis of three immature and mature eggs using 3D renderings shown in (**C**). Scale bar, 50 µm. Statistics, Student's t-test. Linked to Figure 3.

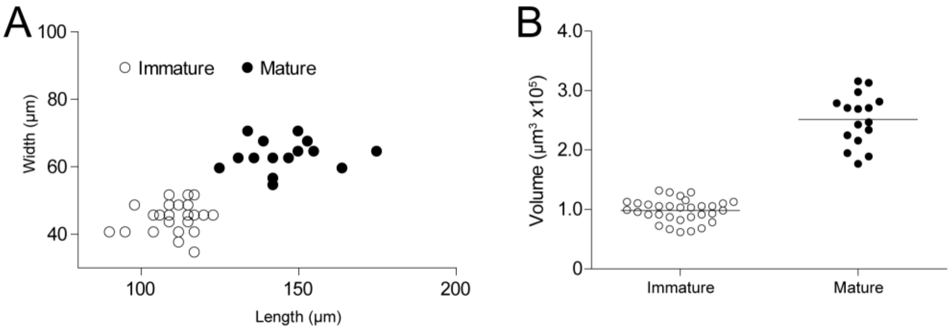

Figure S5. Dimensions and volume of mature and immature eggs
(A) Measurements of *S. mansoni* eggs isolated from mouse livers that were imaged and classified as immature (open circles) or mature (closed circles) based on visual estimate of size and morphology. (B) Egg volumes calculated from egg dimensions in (A). Linked to Figure 5.
